# Supplementary material for: Elaboration of Thermally Performing Polyurethane Foams, Based on Biopolyols, with Thermal Insulating Applications
Source: Polymers (Basel). 2024 Jan 16;16(2):258. doi: 10.3390/polym16020258 (PMC10821512; doi:10.3390/polym16020258)
Supplement: Supplementary file 1 [file polymers-16-00258-s001.zip › polymers-2756691-supplementary.pdf]

Supplementary Information

S1. Supplementary material and methods

S1.1 Description of the process of the production of biopolyols

In the following figure, the different steps, reagents and conditions for the process employed for the production of the biopolyol are showed. The raw material, reagents and products appear in green, the conditions used in each stage of the process are displayed in red, and the different steps are labelled in blue. During the optimization, the amount of raw material that was fed into the reaction was reduced to 120g and so were the solvents (600g). The amount of catalyst employed was kept at 4.5% (w/w) with respect to the solvents. The rest of the conditions are shown in figure SI1.

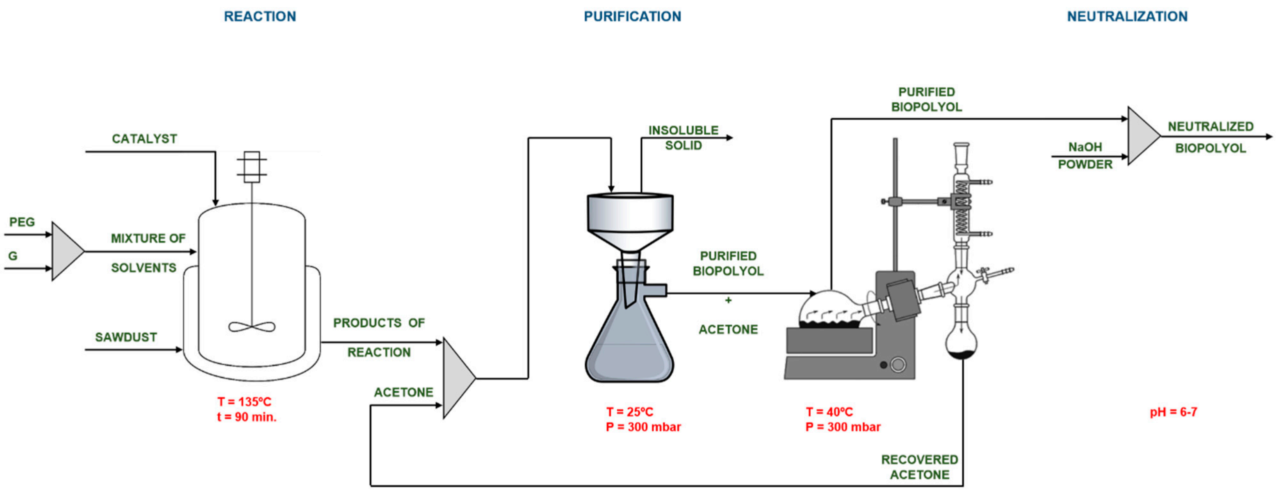

Figure S1. Flow diagram of the process of liquefaction from wood sawdust for the production of biopolyols.

In the first stage (REACTION), the mixture of the solvents with the catalyst and the raw material (wood sawdust) was introduced into the agitated reactor at the depicted conditions. After the reaction time (90 minutes), the products obtained were taken out of the reactor and mixed with an excess of acetone. The acetone decreased the viscosity of the products from the reaction and made the handling easier.

In the second stage (PURIFICATION), the mix of the products from the reaction and the acetone was filtered under a vacuum with a cellulose filter. By means of this process, two separate phases were obtained, namely, insoluble solid (wood sawdust that was not dissolved in the reaction) and the purified polyol with the excess of acetone.

In the final stage (NEUTRALIZATION), the polyol was isolated from the acetone by using a rotatory evaporator. Thereby, it was possible to recover the acetone to be used in other batches for the stage of purification and to collect the purified polyol. This polyol displayed a significantly acidic pH, which could lead to different issues in the production of the polyuretyhanes. For this reason, sodium hydroxide in a powder state was added to neutralize the polyol.

S1.2 Description of the process and stages of the elaboration of the PUFs

In the next figure, a diagram of the process of the elaboration of the PUFs is presented with the different stages, reagents and conditions.

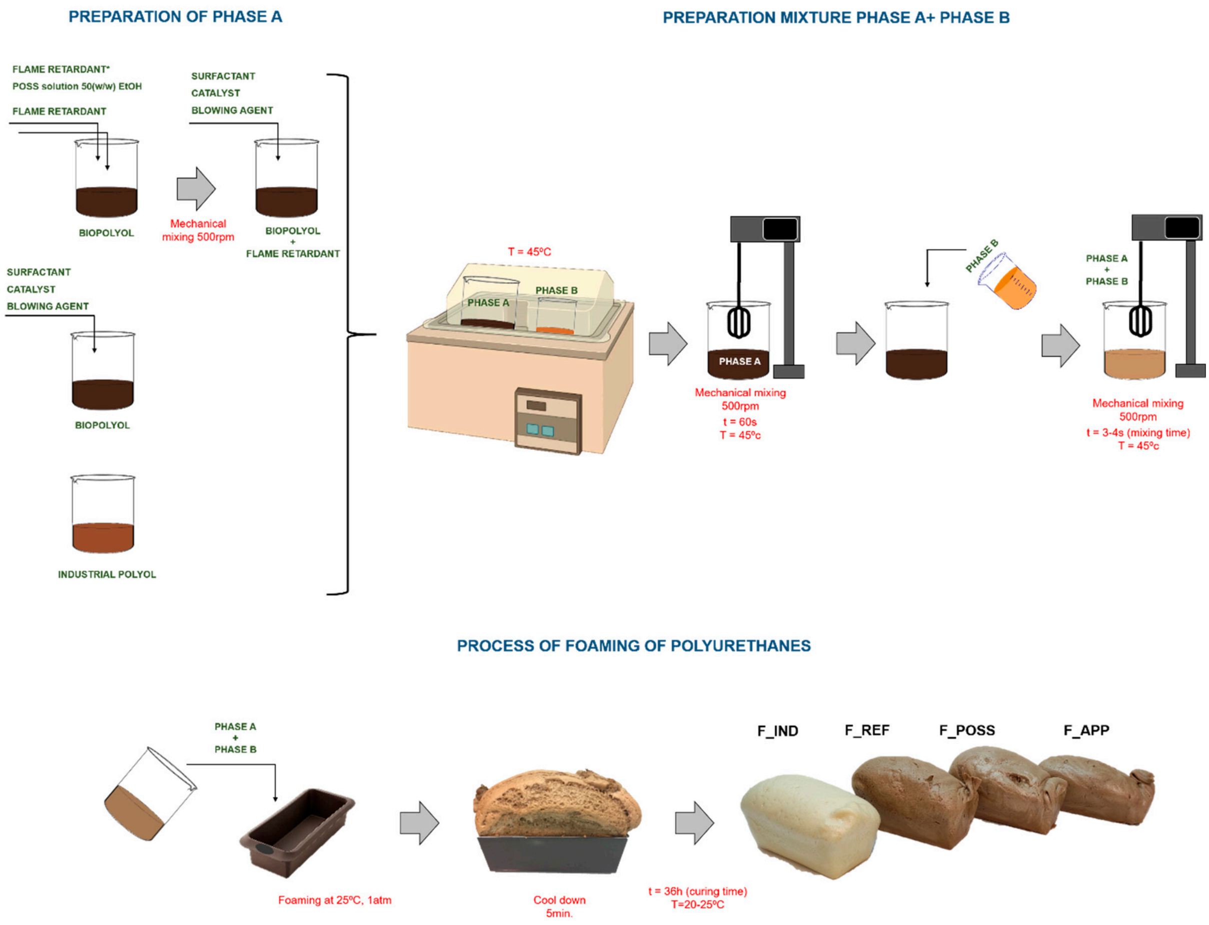

Figure S2. Flow diagram of the process of preparation of the different formulations of PUFs.

In the preparation of phase A, the polyol and the different additives (depending on the formulation) were incorporated into a flask, and they were stirred vigorously. In the case of the industrial formulation, this was not necessary, since phase A was provided by the industrial partner with the polyol and all the additives in the same mixture.

In the preparation of the mixture (phase A+ phase B), phase A (polyol and additives) and phase B (isocyanate) were heated until the desired temperature was reached. At this point, the components in phase A were stirred again for 60 seconds, and then, phase B was added.

In the process of foaming of the polyurethanes, right after phase B was poured onto phase A, the mix was agitated for a brief period of time until a slight color change was detected. Then, the mixture was poured into the silicone mold to foam freely. Once the polyurethane had foamed to its maximum size, it was left cooling down. Next, it was taken out from the mold, and the foams were cured at ambient conditions for 36h.

S2. Supplementary results and discussion

S.2.1 Morphology analysis of the PUFs

In figure S3 a) and b), zoomed-in pictures of the cell morphology of F_POSS and F_APP are shown.

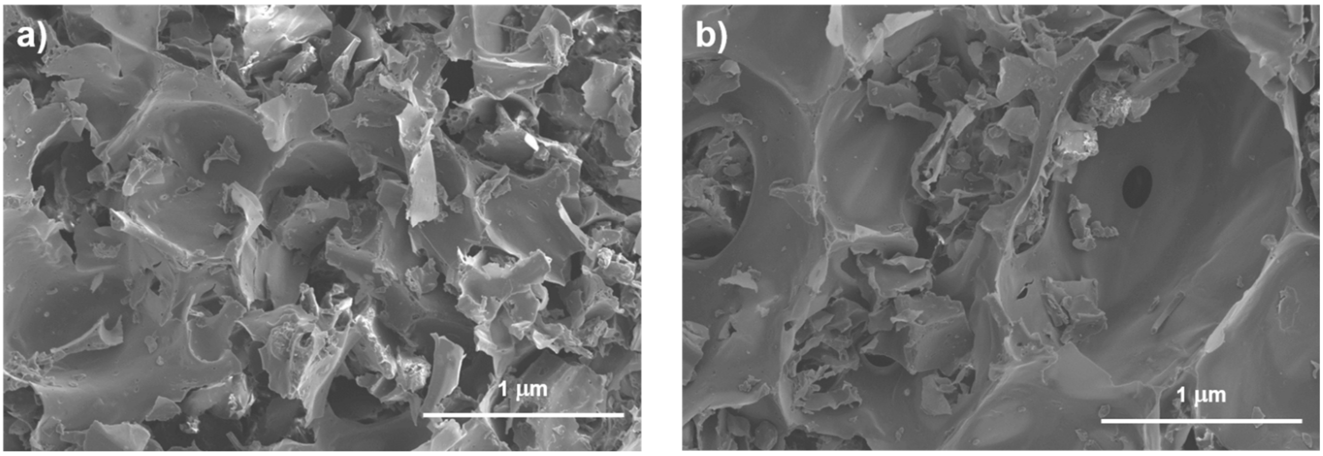

Figure S3. Micrographs with 200 times magnification of the cells in F_POSS (a) and F_APP (b).

S2.2 Fireproofing properties and flame retardance performance of the PUFs

In figure S4, the samples remaining from the UL-94 vertical burning test are displayed.

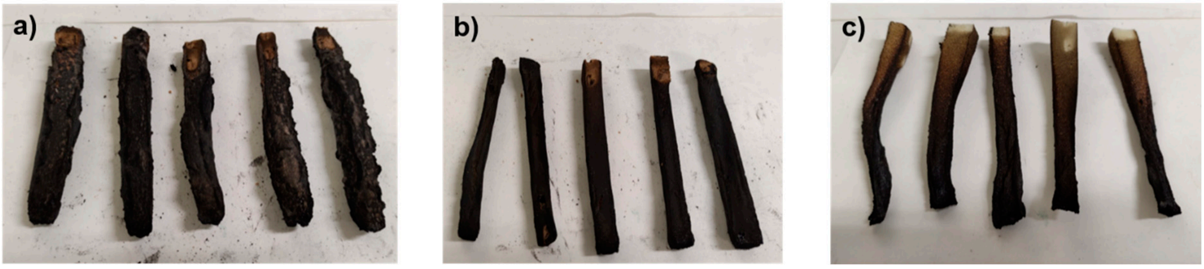

Figure S4. Final state of samples of a) F_POSS, b) F_APP and c) F_IND after burning during UL-94 vertical burning test.
